# Deposition of Diamond Films in a Closed Hot Filament CVD System

**DOI:** 10.6028/jres.100.004

**Published:** 1995

**Authors:** Guan-Ren Lai, E. N. Farabaugh, A. Feldman, L. H. Robins

**Affiliations:** National Institute of Standards and Technology, Gaithersburg, MD 20899-0001

**Keywords:** chemical vapor deposition, closed system, diamond films, Raman, scanning electron microscopy, x-ray diffraction

## Abstract

A closed system hot filament chemical vapor deposition (CVD) reactor has been used to deposit diamond films on silicon substrates. A fixed charge of hydrogen gas is fed into the deposition system until the desired deposition pressure level is reached. A solid graphite cylindrical rod held above the tungsten filament was the carbon source. System parameters for diamond film growth have been determined. The diamond structure of the films has been verified by x-ray diffraction (XRD). Morphology typical of CVD diamond films has been observed in scanning electron microscopy (SEM). The quality of the diamond films has been evaluated by micro-Raman spectroscopy.

## 1. Introduction

The unique physical, chemical and mechanical properties of diamond make it attractive for many applications such as wear resistant coatings, cutting tools, and heat spreaders for electronic devices. Several methods are used successfully to produce diamond films such as hot filament chemical vapor deposition (CVD) [1], electron assisted CVD [2], dc discharge CVD [3], RF discharge CVD [4], microwave plasma-assisted CVD [5], and laser-assisted deposition [6]. Almost all these approaches employ feed gases that are constantly being replenished. A feed gas mixture made up of hydrogen, a hydrocarbon gas, and sometimes oxygen flows into the deposition chamber and a vacuum pump is employed to maintain a constant pressure. Some large deposition systems recirculate the gases, however, using large pumps in the process.

In most deposition processes, atomic hydrogen is critical to the formation of the diamond *sp*^3^ bond. Atomic hydrogen also attacks the graphitic *sp*^2^ bond, removing graphite that tends to form with the diamond. However, hydrogen is not consumed in the diamond deposition process. Thus, hydrogen is wasted when it is pumped out of the deposition chamber during the deposition process. By using a fixed quantity of hydrogen to produce diamond films in a closed system we can minimize the use of hydrogen, thus reducing the cost of producing CVD diamond films. The deposition process is also safer with a closed system because the dissipation of large quantities of explosive hydrogen gas into the atmosphere is eliminated.

In this paper, we report on the deposition of diamond in a closed hot filament reactor which contains a fixed quantity of hydrogen and a graphite rod as the carbon source. The hot filament produces atomic hydrogen which reacts with the carbon source to produce gaseous hydrocarbon species which are precursor gases needed to produce diamond. These precursor species react with the atomic hydrogen at the substrate surface to form diamond.

## 2. Experimental

**Fig. 1:**
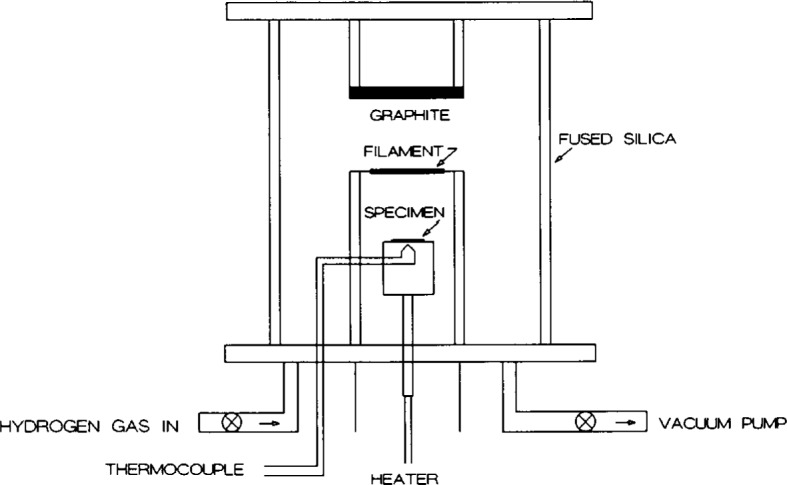
The schematic diagram of a closed hot filament CVD reactor.

Figure 1 is a schematic diagram of our closed hot filament CVD reactor. The system uses a quartz cylinder as the reaction chamber. The chamber contains a tungsten filament, a heated substrate holder, a carbon rod source, a sealable gas inlet and pumping port, and a thermocouple temperature sensor. No hydrocarbon gases are fed into the system. The only gas introduced into the operating chamber is hydrogen. The tungsten filament is located at the center of the chamber. The specimen substrate is placed on the heater block below the filament. A solid graphite rod placed above the filament and parallel to it is the carbon source.

The chamber is initially evacuated with a mechanical pump. When the desired base pressure is reached, the pumping port valve is closed and hydrogen gas is then allowed to flow into the chamber to the desired pressure. The gas inlet valve is then closed.

It is necessary that there be no leaks in the system. The system was leak tested with a helium leak tester and found to be sufficiently leak tight. The substrate temperature is measured with a thermocouple mounted in the heater block directly under the substrate. The temperature of the filament is determined with a disappearing filament optical pyrometer.

Two types of substrates were used in this study in order to determine whether the growth rate of diamond on bare substrates was different from the growth rate on diamond coated substrates. The substrates used were polished single crystal {111} silicon wafers and single crystal {111} silicon wafers that have been previously coated with a layer of diamond. All specimens were 1 cm square. The uncoated silicon substrates were pretreated by rubbing with 0.5 μm diamond paste, washing with ethyl alcohol, and then drying in air. The coated substrates were produced in a flowing gas hot filament reactor.

Initially, a five-turn helical tungsten wire was used as a filament. However, such filaments tend to sag during the deposition and the filament to substrate distance is not well defined. Therefore, we modified the filament holder so that a straight wire filament under tension could be used. This eliminated the sagging problem and made it possible to maintain a uniform spacing between the substrate and the filament. The parameters for diamond film deposition are listed in Table 1.

## 3. Results and Discussion

Diamond was grown successfully in the closed chamber under a variety of conditions. The growth of diamond in a closed reactor is envisioned as follows. The hot filament produces atomic hydrogen which serves two functions. First, the atomic hydrogen reacts with the graphite to produce gaseous hydrocarbon species that are precursors to the growth of diamond. Second, the atomic hydrogen reacts with the hydrocarbon gases at the substrate surface to produce diamond. All of the transport processes are considered to take place by diffusion although natural convection might occur. The rate of hydrocarbon gas production is expected to decrease with increasing distance of the graphite rod from the filament. A steady state condition is expected in which the rate of hydrocarbon production from etching of graphite is balanced by the rate of diamond deposition. Whether our not our depositions have reached the steady state condition has not yet been evaluated.

**Fig. 2:**
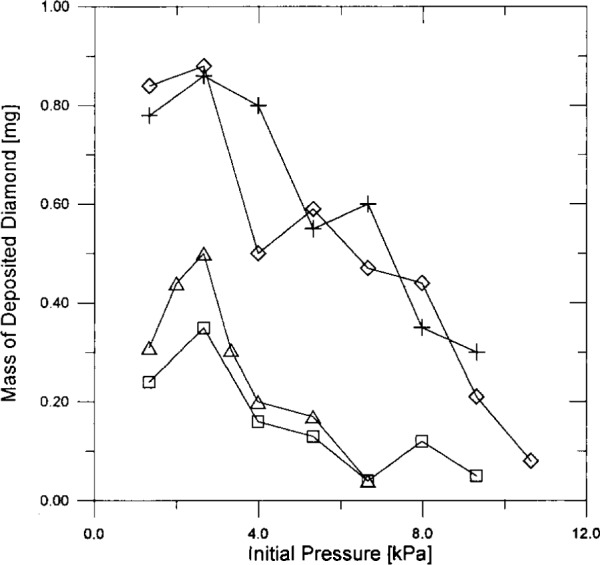
The mass gain vs initial chamber pressure for diamond films grown for a 4 h and *D* = 7 mm. ♢, diamond film on Si, five-turn helical filament; +, diamond film on diamond coated Si, five-turn helical filament; □, diamond film on Si, straight wire filament; Δ, diamond film on diamond coated Si, straight wire filament.

Figure 2 shows the mass gain vs chamber pressure for diamond films grown over a 4 hour period using a filament-rod spacing, *D*, of 7 mm. In these mass measurements we are assuming that the deposited carbon species is diamond. There is some non-diamond bonded carbon also deposited (as can be seen in the Raman spectra presented later in this paper) but it is assumed that the amount is small compared to deposited diamond. Measurements of the mass of similar samples have shown that a standard deviation of .02 mg can be expected. The deviation is based on the instrumental weight measurement and specimen sampling. It is clearly seen that in all cases there is maximum mass gain at a deposition pressure of 2.7 × 10^3^ Pa (20 Torr). The mass gain with the five-turn helical filament is larger than the mass gain with the straight wire filament by factors of perhaps 2 to 3. We believe this may be partially due to the longer length of the helical filament. If we assume that the production rate of reactive diamond precursor species, such as CH_3_^+^, CH_2_^++^, H^+^, etc., increases with increasing filament surface area, then this result is reasonable. The computed length of the helical filament is about 1.4 times the length of the straight filament. Thus, the observed larger growth rate with the helical filament is not strictly proportional to the filament length. This is not surprising in view of previous results that related diamond growth rate with filament turn density reported previously [7]·

The growth rate on silicon substrates appears to be essentially the same as the growth rate on diamond. There is some indication that in the straight wire depositions, the growth rate on diamond coated substrates was slightly higher than growth rates on silicon substrates.

**Fig. 3:**
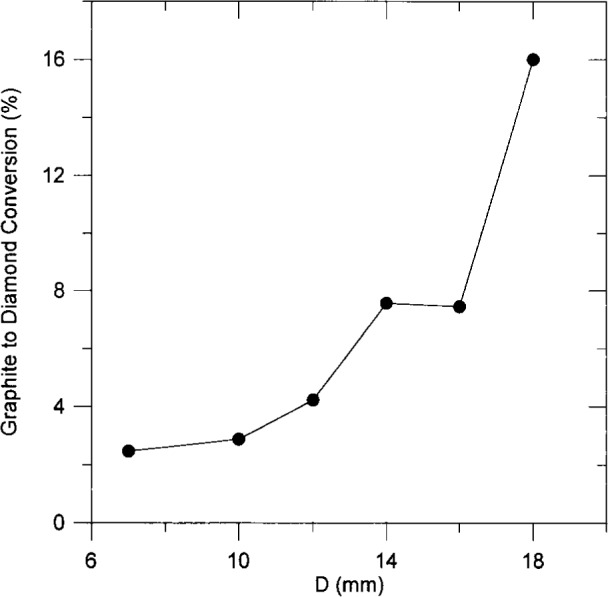
Mass loss of the graphite rod and mass gain of diamond vs filament-graphite rod spacing. ♢, graphite mass loss; □, diamond mass gain.

Figure 3 compares the diamond mass increase during deposition with the mass decrease of the graphite rod as a function of the graphite to filament spacing, *D*. The upper curve shows the mass loss of graphite during a 4 h diamond deposition; the lower curve shows the mass gain of the diamond film. When the graphite rod is closest to the filament mass removal rate is relatively high. As *D* increases, the mass removal rate drops off sharply. In contrast, the diamond mass increase varies only slowly with *D*. As expected, the deposition rate gradually decreases as the spacing between the filament and rod increases.

**Fig. 4:**
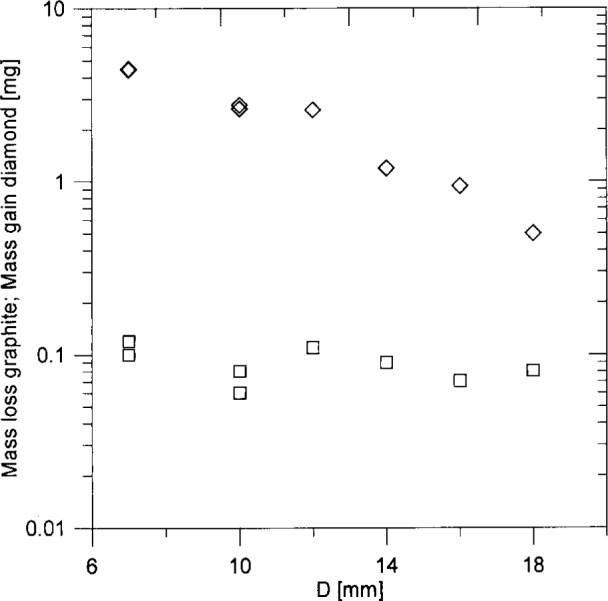
Percentage conversion of graphite to diamond vs filament-graphite rod spacing.

Figure 4 shows the fraction of etched graphite converted to diamond as a function of *D*. It is clearly seen that the conversion efficiency increases with increasing *D*.

**Fig. 5:**
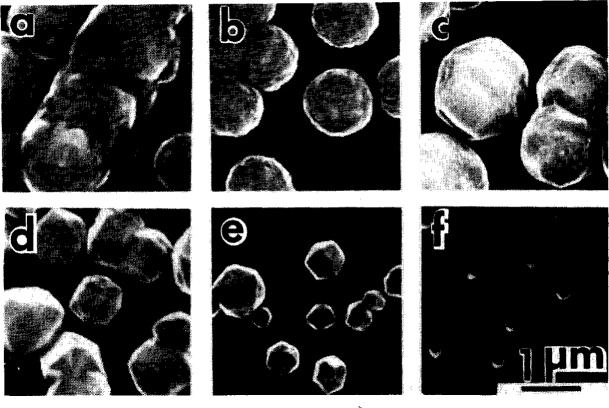
Scanning electron micrographs of diamond particles grown on (111) Si in a closed hot filament CVD reactor for different values of the filament-graphite rod spacing *D*. (a) *D* =7 mm; (b) *D* = 10 mm; (c) *D* = 12 mm; (d) *D* = 14 mm; (e) *D* = 16 mm; (f) *D* = 20 mm.

We examined several samples in a scanning electron microscope (SEM). The micrographs are shown in Fig. 5. Each micrograph shows the morphology of a diamond coating with different filament to graphite spacings, *D*, but the same filament to substrate spacing of 4 mm. The values of *D* corresponding to each micrograph are: 5(a), *D* = 7 mm; 5(b), *D* = 10 mm; 5(c), *D* = 12 mm; 5(d), *D* = 14 mm; 5(e), *D* = 16 mm; 5(f), *D* =20 mm. Each of the depositions resulted in diamond particle growth; no continuous films were formed within the 4 hour depositions. The surface morphology of the particles is seen to vary with *D*. The particle morphology evolves from unfaceted, poorly defined surface features, Fig. 5(a), to sharply faceted morphology on very small individual particles, Fig. 5(f). This sequence corresponds to increasing *D*. If we assume that the best quality diamond corresponds to the sharpest crystal morphology, then the diamond particle quality improves with increasing *D*. However, that growth rate, as shown by the particle size in the micrographs, shows a corresponding decrease.

When the graphite etch rate is high (smaller *D*), it is expected that the hydrocarbon concentrations in the chamber are at a high level; thus, the diamond quality is expected to be relatively poor. When the graphite etch rate is low (larger *D*), we expect that the hydrocarbon gas concentrations in the chamber are at a low level. In this case the diamond quality is expected to be relatively good. This interpretation is consistent with well-known observations in conventional diamond growth; the quality of the diamond decreases with increasing methane fraction in the feed gas when hydrogen/methane mixtures are used.

**Fig. 6:**
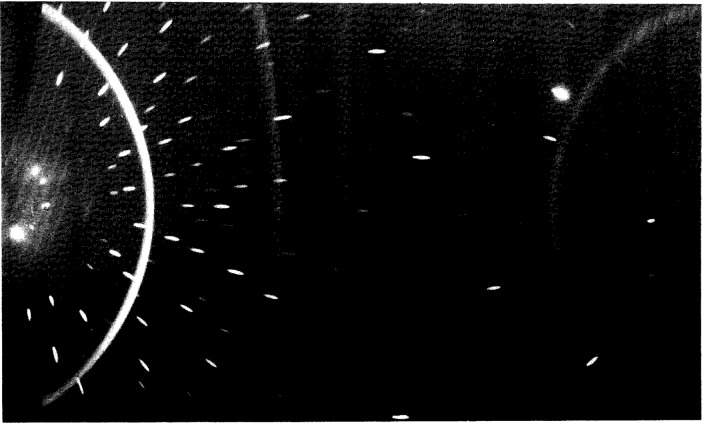
Read camera x-ray diffraction pattern of a diamond film deposited in a closed hot filament CVD reactor on a (111) Si substrate. The deposition time was 65 h and the filament-graphite rod spacing was 16 mm.

**Fig. 7:**
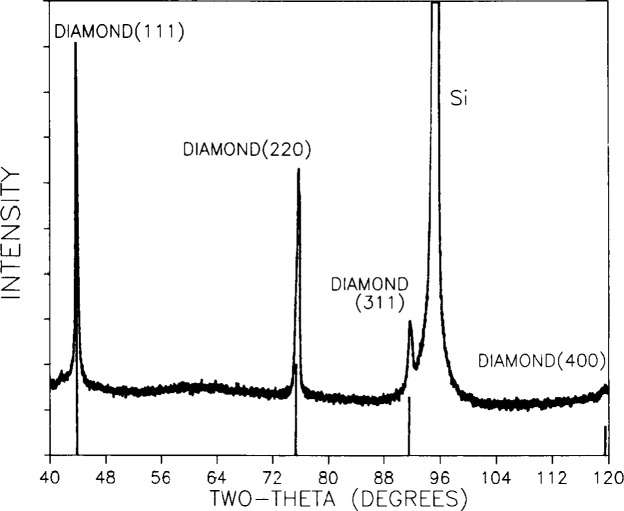
X-ray diffraction pattern of a diamond film deposited on a (111) Si substrate. The deposition time was 65 h and the filament-graphite rod spacing was 16 mm.

**Fig. 8:**
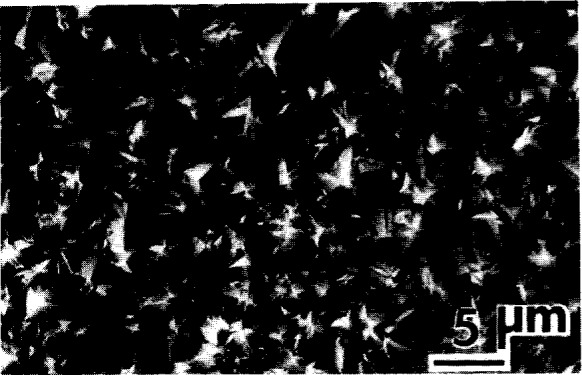
Scanning electron micrograph of the diamond film used to produce the x-ray patterns shown in Fig. 6 and 7. The deposition time was 65 h and the filament-graphite rod spacing was 16 mm.

The presence of the diamond phase in the deposited films was verified by x-ray diffraction (XRD) with a Read camera [8] and with powder diffractometry. Figure 6 shows a Read camera diffraction pattern and Fig. 7 shows a powder diffraction pattern obtained from the same diamond film. Figure 8 shows a SEM micrograph of the surface of this film. The diamond film was deposited for 65 h with *D* = 16 mm and a 4 mm spacing between the filament and substrate. In Fig. 6 all five of the rings can be attributed to diamond diffraction. The Laue spots in the figure are Laue spots originating from the single crystal Si substrate. The diamond peaks in Fig. 7 are indicated by the markers and the large peak at 2*θ* = 95° is due to (333) diffraction from the Si substrate.

**Fig. 9:**
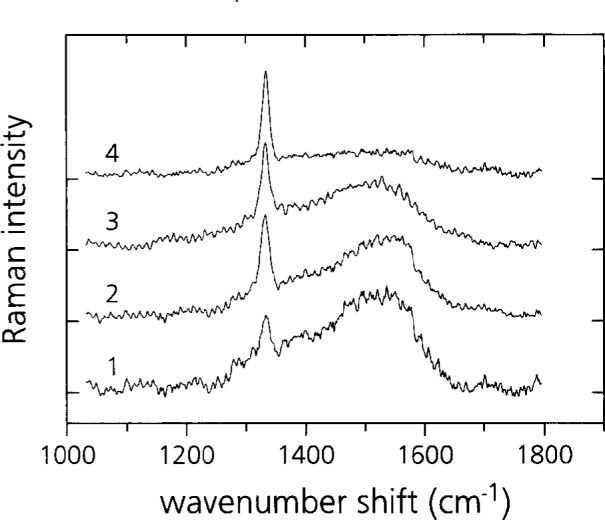
Raman spectrum from four continuous diamond films grown with different filament-graphite rod spacing, *D*. (1) *D* =7 mm, 88 h deposition time; (2) *D* = 16 mm, 88 h deposition time; (3) *D* = 16 mm, 65 h deposition time; (4) *D* = 18 mm, 88 h deposition time.

Figure 9 shows the Raman spectra of four diamond films over a wavenumber range 1100 cm^−1^ to 1800 cm^−1^. The luminescence background has been subtracted in these spectra. An Ar^+^ ion laser operating at the wavelength 514.5 nm was the excitation source; the laser power was 80 mW and the laser spot size was ~100 μm. Spectra 1–4 were obtained from films prepared as follows: (1) *D* =7 mm, 88 h deposition time; (2) *D* = 16 mm, 88 h deposition time; (3) *D* = 16 mm, 65 h deposition time; (4) *D* = 18 mm, 88 h deposition time. The filament-substrate spacing was 4 mm in all cases. Two features are present in all of the spectra, the peak from diamond bonded carbon at 1332 cm^−1^ and the band from non-diamond bonded (*sp*^2^) carbon at 1550 cm^−1^. The spectra indicate that the quality of the diamond improves as *D* increases. (Compare spectra 1, 2, and 4.) The *sp*^2^ band is weakest for the diamond grown with *D* = 18 mm and strongest for diamond grown with *D* =7 mm, the spacing that produced the fastest etching rate of the carbon rod. The results supports the conclusions reached from the morphology examinations discussed above. It is also to be noted that the longer of the two depositions, 2 and 3, carried out with *D* = 16 displayed the stronger *sp*^2^-bonded carbon band. However, more data is needed to verify a possible trend of longer depositions producing poorer quality diamond for a fixed deposition geometry.

## 4. Summary

We have demonstrated that diamond films can be produced in a closed hot filament CVD system. The deposition of the diamond phase was verified by XRD, Raman spectroscopy, and SEM. The closed system differs from the conventional hot filament CVD system in that no flowing gases are used during the deposition. The source of carbon is a carbon rod mounted above the tungsten filament. Diamond particles and films were produced. The quality and growth rate of the diamond is dependent on filament-graphite rod spacing. Spacings of 14 mm to 16 mm resulted in well faceted diamond. Larger spacing produced better faceted diamond but at much reduced growth rates. Smaller spacings yield higher growth rates but poorer quality diamond. The successful deposition of diamond in a closed system promises to result in lower diamond production costs. In addition, the safety of the deposition process is improved by minimization of the amount of hydrogen used during the deposition and discharged into the atmosphere.

## Figures and Tables

**Table 1:** Parameters for closed system diamond film deposition

Base pressure	<0.13 Pa (10^−3^ Torr)
Operating pressure	1.3 kPa to 10.6 kPa (10 Torr to 80 Torr)
Filament material	Tungsten wire, 0.125 mm diameter
Filament configurations	Five-turn helix ~2 cm long 1.25 mm mean coil diameter straight wire ~2.5 cm long
Filament temperature	1700 °C to 2000 °C
Distance, filament to substrate	2 mm to 4 mm
Distance, filament to carbon rod	7 mm to 20 mm
Substrates	{111} silicon wafer, 1 cm square rubbed with 0.5 μm diamond paste, rinsed with ethanol diamond coated {111} silicon wafer, 1 cm square rinsed with ethanol
Substrate temperature	750 °C
